# The evaluation of a fast and simple pesticide multiresidue method in various herbs by gas chromatography

**DOI:** 10.1007/s11418-013-0777-9

**Published:** 2013-05-14

**Authors:** Bożena Łozowicka, Magdalena Jankowska, Ewa Rutkowska, Izabela Hrynko, Piotr Kaczyński, Jan Miciński

**Affiliations:** 1 Plant Protection Institute-National Research Institute, Regional Experimental Station, Laboratory of Pesticide Residues, Chelmonskiego 22, 15-195 Bialystok, Poland; 2 Faculty of Animal Bioengineering, University of Warmia and Mazury in Olsztyn, Oczapowskiego 5/150, 10-719 Olsztyn, Poland

**Keywords:** Pesticide, Herb, Gas chromatography, Multiresidue method, MSPD, LSE

## Abstract

In this study two analytical methods, one based on matrix solid phase dispersion (MSPD) and the other on liquid–solid extraction (LSE), coupled with gas chromatography, were evaluated and used to determine the presence of 163 pesticides (6 acaricides, 62 fungicides, 18 herbicides and 77 insecticides) in various herbs. Both methods were optimized considering different parameters (sample to sorbent mass ratio, extracting solvent, sorbents for clean-up step, etc.). The results of these validated sample preparation procedures were compared. Under optimum conditions, the mean recoveries obtained were in the range of 70–119 % for MSPD for most pesticides and 70–118 % for LSE, but with several exceptions. Precision values, expressed as relative standard deviation (RSD), were ≤16 % for MSPD and <18 % for LSE. Correlation coefficients were higher than 0.99254 for both methods. LODs (limits of detection) and LOQs (limits of quantification) for MSPD were within the ranges of 0.003–0.03 and 0.005–0.04 mg/kg, respectively. The data demonstrate that the MSPD method was successfully used for the analysis of 163 pesticides in the following herbs: chamomile (*Matricaria chamomilla* L.), linden (*Tilia*), lungwort (*Pulmonaria* L.), melissa (*Melissa* L.), peppermint (*Mentha piperita* L.) and thyme (*Thymus vulgaris* L.). This paper indicates the potential of MSPD for qualitative and quantitative analysis of pesticide residues. This method was therefore validated at three spiking levels (the first ranging from 0.005 to 0.05 mg/kg, the second from 0.05 to 0.5 mg/kg and the third from 0.25 to 2.5 mg/kg) and applied to real samples (*n* = 15). MSPD proves to be a simple, fast and very useful multiresidue method and can be recommended for routine pesticide monitoring studies in various herbs.

## Introduction

Herbs play an important role in our health and our food and have a variety of culinary and medicinal uses. Although herbs have been in use in the human diet and traditional medicine since antiquity, they have recently become the center of attention of the nutrition-science world because of their potential health benefits and detoxification properties. There are many herbal benefits: they have hypotensive or antihypertensive effects [1, 2] and contain unique anti-oxidants [3], essential oils, vitamins, phytosterols and many other plant-derived nutrients, which help the immune system defend the body against viruses, toxins, bacteria and other germs [4].

In general, medicinal plants and herbal materials may be found with various kinds of microbial contaminants, of which bacterial and fungal infections are regarded as the most common [5]. Beside biological contaminants, herbs may be contaminated by toxic chemical substances such as mycotoxins, heavy metals, pesticides and deposited pesticide residues.

Similar to other crops, herbal plants are attacked by insects and diseases both in the field and during storage, and therefore pesticides are widely used for their protection. Attention is focused on pesticide contamination due to its high toxicity and persistence in the environment. Although the use of organochlorine pesticides (OCPs) has been restricted or forbidden by legislation for many years, these compounds are still being detected [6]. Pesticide contaminants may be related to the origin of these herbal plants, such as when they are growing in a contaminated environment, e.g. in soil where banned pesticides, such as DDT [7], have been deposited for many years. During the growing and post-harvest periods herbs can be protected against agrophages through the controlled use of plant protection products (insecticides and herbicides) [8]. This is the first source of pesticide residues. The second source is the uncontrolled application of biopesticides against mosquitoes on large areas of forests.

It is well known that there are many contaminants and residues that may cause harm to the consumers of herbal medicines [9]. Herbal materials and medicinal plants are also often used as food, functional food, and nutritional and dietary supplements. Thus, medicinal plants and herbal products must be safe for patients and consumers. It is, therefore, essential to establish a convenient quality control method to assure the safety of herbal products.

To prevent and screen for pesticide residues and to ensure safety and conformity of quality standards, medicinal herbs and herbal products should be included in the appropriate regulatory framework. Herbs are classified as foodstuffs of plant origin by Regulation (EC) 396/2005 [10] (a herb can be a leaf, flower, stem, seed, root, fruit, bark or any other part of a plant) and as herbal drugs according to the European Pharmacopoeia [11].

In order to ensure consumer safety, authorities in Europe have set maximum residue limits (MRLs) for some pesticides in herbs [12]. Because of the widespread use of plant protection products to protect herbs during cultivation, control of their residues has become a necessity. In cases when herbs are used as medicinal drugs there is a need of guarantee in a form of certificate for pesticide residues.

The analytical determination of pesticides in herbs with an unidentified pesticide treatment history is a formidable task, because it involves the identification and quantification of several hundred possible single or combinations of compounds in the presence of complex matrices.

Only a few analytical methods for the determination of pesticide residues in herbs have been described in the recent literature and they are limited to selected compounds or groups [13–15]. In the case of herbs, no more than 30 pesticides were included in a single method. Therefore, it was considered desirable to devise a novel procedure that would allow for screening a much broader range of pesticides (approximately 163) to assure the production of good quality herbal products. The published studies are based on Soxhlet extraction [16], microwave-assisted extraction (MAE) [13] or on the QuChERS method [15], and are very often followed by gas chromatography–mass spectrometry (GC–MS) [17, 18]. Some research works have studied pesticide residues in herbal material and were mainly based on surveying and monitoring market samples [16].

Herb sample preparation is a crucial step in pesticide residue analysis. In recent times, research has been focusing on those methods which allow for reduction of the organic solvent, and the elimination of the additional sample clean-up and pre-concentration steps before chromatographic analysis [19]. The complexity of the herb matrix is due to the presence of phenolic compounds, carotenoids, chlorophyll and essential oils [20]. In order to eliminate the effects of interference and to avoid the matrix effect it is necessary to develop a sensitive method.

To the best of our knowledge, no analytical method has been developed able to simultaneously determine multi-pesticide residues in herbs like linden, lungwort, melissa and peppermint using matrix solid phase dispersion (MSPD).

Matrix solid phase dispersion has been used for performing the extraction of a variety of matrices from a number of compounds, e.g. caffeine in green tea leaves [21], rutin in *Sambucus nigra* L. (elderberry) [22], polybrominated diphenyl ethers and polychlorinated biphenyls in biota samples [23], phenolic compounds in fruit-green tea [24], free fatty acids in chocolate [25] and pesticides in fruits and vegetables [26, 27], soil [28] or bees [29]. However, little is known about the application of MSPD as a sample preparation method for the analysis of various groups of pesticides in herbs. Previous papers adopting this extraction approach refer to only a few pesticides in herbs by GC [30–32].

The main objective of this work was to optimize the process of preparation, extraction and purification of herbal samples using MSPD and liquid–solid extraction (LSE) for qualitative and quantitative analysis of a wide spectrum of pesticide residues.

The analytical novelty of this work is the validation of an efficient, sensitive, interference-free, fast and simple MSPD method that would allow determination of over 160 pesticides representing a wide range of physicochemical properties in complex herb matrices. In addition, this paper shows the potential of MSPD as a convenient method for the analysis of a wide range of pesticides in various herbs.

## Experimental

### Chemicals and materials

Acetone, acetonitrile, dichloromethane, diethyl ether, *n*-hexane and methanol for pesticides residue analysis were provided by J.T. Baker (Deventer, Holland), and Florisil (60–100 mesh), anhydrous sodium sulfate, Celite and octadecyl silica gel C_18_ (200–400 mesh) were purchased from Fluka (Seelze-Hannover, Germany). Silica gel (230–400 mesh) and neutral aluminum oxide (0.063–0.200 mm) were obtained from Merck (Darmstadt, Germany). Sorbents were activated at 600 °C, with the exception of the neutral aluminum oxide which was activated at 130 °C. Deactivated sorbents were prepared by adding the appropriate amount of distilled water to activated sorbents (for preparation of 5 % neutral aluminum oxide and 4 % Florisil, 5 ml and 4 ml of water was added to obtain 100 g, respectively).

The 163 pesticide standards were purchased from Dr. Ehrenstorfer Laboratory (Germany). The purities of the standard pesticides ranged from 95 to 99.8 %. Each stock solution at various concentrations was prepared in acetone and stored at 4 °C for further dilution. Multicompound standard working solutions (M1–M4, each containing about forty active substances) were prepared by dissolving 0.2–4.0 ml of each stock solution in an *n*-hexane/acetone (9:1, v/v) mixture to give a final concentration range of 0.05–1.0 μg/ml. The stock and working solutions were stored in completely filled vials closed with parafilm at −20 °C until analysis.

### Samples

The following herbs were used in the experiment: chamomile (*Matricaria chamomilla* L.), linden (*Tilia*), lungwort (*Pulmonaria* L.), melissa (*Melissa* L.), peppermint (*Mentha piperita* L.) and thyme (*Thymus vulgaris* L.). All of them were cultivated in north-eastern Poland (cultivation year 2010). These samples were used for blanks, fortified samples for recovery assays and matrix-matched standards for calibration in the comparison of methods. About 1 kg portions of the herbs were air-dried (at a temperature of approximately 40 °C), cut and ground. Samples were stored until the moment of extraction at 4 °C, then the plant material was ground and its portion was used in the applied sample preparation procedure. Samples (*n* = 15) of herbs for the monitoring study were purchased from local producers: chamomile (*n* = 1), linden (*n* = 3), lungwort (*n* = 3), melissa (*n* = 3), peppermint (*n* = 3) and thyme (*n* = 2).

### Sample preparation

#### Matrix solid phase dispersion—MSPD (Procedure 1)

Two grams of the ground herb sample were put in a mortar with 4 g of solid support (Florisil), and manually blended using a pestle to obtain a homogeneous mixture. After homogenization, the blend was quantitatively transferred with a spatula to a glass macro column packed with anhydrous sodium sulfate (5 g), octadecyl C_18_ (1 g) and silica gel (2.5 g). The absorbed analytes were then eluted using 25 ml acetone/methanol (9:1, v/v).

#### Liquid–solid extraction—LSE (Procedure 2)

Two grams of the ground herb sample were weighed in an Erlenmeyer flask. Extraction was carried out by placing the sample with 50 ml of hexane/diethyl ether/acetone (1:2:2, v/v/v) as an extracting solvent on a rotary shaker (Ika Shaker KS 501 digital) at high speed (2500 rpm) for 30 min. The extract was filtered through a filter with 5 g of Celite and 5 g of anhydrous sodium sulfate, then a 20 ml portion of hexane/diethyl ether/acetone (1:2:2, v/v/v) was added and shaken for another 30 min. Extracts were then combined in the same flask and evaporated until dry using a rotary evaporator at a temperature of about 40 °C. The dry residue was then dissolved in 2 ml of hexane/acetone (9:1, v/v). The extract was cleaned on a chromatography column containing sodium sulfate (2 g), 5 % neutral aluminum oxide (2.5 g) and 4 % Florisil (2 g) using 30 ml hexane/dichloromethane (7:3, v/v).

**Fig. 1:**
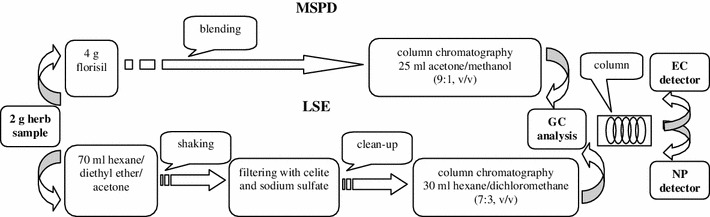
Sample preparation procedures and dual system of detection

The extracts obtained from Procedures 1 and 2 were evaporated until dry using a rotary evaporator at a temperature of approximately 40 °C and the dry residue was re-dissolved in 2 ml of hexane/acetone (9:1, v/v) and then transferred to 2 ml vials for further GC-NP/EC analysis. The stages of both preparation procedures are shown in Fig. 1.

#### Preparation of spiked herb samples

For both procedures, matrix-matched standards were prepared at concentration levels ranging from 0.05 to 0.5 mg/kg. Blank herb samples (previously tested for the absence of pesticide residues) were used for fortification experiments. Spiked samples were prepared by adding an appropriate volume of spiking solution to exactly weighed portions of milled plant material (2 g) and left for 1 h (to allow pesticide absorption by the sample). Sample preparation was carried out using the two techniques, MSPD and LSE. The main purpose of this step was to calculate the average of the recovery percent of the investigated pesticides through both extraction techniques.

### Chromatographic analysis

Pesticide analysis was performed using an Agilent 7890 A gas chromatograph (Santa Clara, CA, USA) equipped with an automatic split–splitless injector Model HP 7683, a ^63^Ni micro-electron capture detector (μEC) and a nitrogen phosphorous detector (NP). The flux at the end of the GC column was divided into two branches by means of a “Y” press-tight connector connected at one end to the GC column and at the other to the two detectors (Fig. 1). Chemstation chromatography manager data acquisition and processing system (Hewlett-Packard, version A.10.2) was used. Chromatographic separation was performed on an Agilent HP-5 column (30 m, 0.32 mm I.D., 0.25 μm film thickness; Little Falls, DE, USA). When positive peaks of pesticides were detected above LODs, the results were confirmed by analysis on the different polarity column. The DB-35, a midpolarity column (35 %-phenyl-methylpolysiloxane) with low bleed (30 m–0.32 mm I.D., 0.5 μm film thickness) supplied by Agilent (Little Falls, DE, USA), was found ideal for conformational analysis. The operating conditions for GC analysis are given in Table 1. Quantification was performed by comparing the heights of peaks obtained in samples with those found in standards (±0.005 min for positive match).

**Table 1:** Conditions for the injection and GC analysis

Injection mode	EC detector		NP detector	
Column	HP-5	DB-35	HP-5	DB-35
Injector temperature program	210 °C	210 °C	210 °C	210 °C
Carrier gas (flow-rate)	Helium 3.0 ml/min	Nitrogen 1.9 ml/min	Helium 3.0 ml/min	Nitrogen 1.9 ml/min
Detector temperature	300 °C	300 °C	300 °C	300 °C
Make up gas (flow-rate)	Nitrogen 57 ml/min	Nitrogen 60 ml/min	Nitrogen 8 ml/min, hydrogen 3.0 ml/min, air 60 ml/min	Nitrogen 8 ml/min, hydrogen 3.0 ml/min, air 60 ml/min
Splitless period (min)	2	2	2	2
Oven temperature program	120–190 °C at 16 °C/min, 230 °C at 8 °C/min to 285 °C at 18 °C/min (13 min)	120–190 °C at 13 °C/min, 240 °C at 8 °C/min to 295 °C at 16 °C/min (15 min)	120–190 °C at 16 °C/min, to 230 °C at 8 °C/min to 285 °C at 18 °C/min (13 min)	120–190 °C at 13 °C/min, 240 °C at 8 °C/min to 295 °C at 16 °C/min (15 min)
Injection volume of final extract (μl)	2	2	2	2
Total time for analysis (min)	25.431	30.070	25.431	30.070
Equilibration time (min)	2	2	2	2

### Validation of method

Blank samples of six different herbs were used to validate the applied methods in accordance with Document SANCO [33].

#### Calibration curve and linearity

Calibration standards for the analysis of pesticides were prepared in a matrix solution (by adding respective spiking solutions to a blank herb matrix) to produce final concentrations between 0.005 and 2.5 mg/kg. Linearity was determined from the coefficients of determination (*R* ^2^).

#### Precision and accuracy; LOD and LOQ

Repeatability (precision) was calculated for five consecutive days using three replicates of three different concentration levels. Precision and accuracy were evaluated by performing recovery studies of each extraction technique and are expressed as relative standard deviation (RSD, %) and mean recovery, respectively. The limits of detection (LOD) and quantification (LOQ) were calculated using signal-to-noise ratio (S/N) criteria in all cases; LOD = 3 S/N and LOQ = 10 S/N.

#### Recovery study

Samples without pesticides were used for fortification experiments. Recovery data was obtained at three different concentrations within the range in the matrix. Blank samples were spiked through the addition of an appropriate volume of a mixture of standard pesticide solution, then the sample was left for 1 h to allow pesticide absorption. The samples were then prepared according to Procedures 1 and 2 described above.

#### Estimation of uncertainty

The actions performed during the uncertainty estimation of the analytical result were in accordance with the Guide to the Expression of Uncertainty in Measurement [34]: defining the measuring procedure and determining the measured value; developing a mathematical model to be used for calculating analytical results based on the measured parameters; finding values for all possible parameters that can influence the final results, and estimating the associated standard uncertainties; applying the law of propagation of uncertainty in order to calculate the combined standard uncertainty of the final results. The combined standard uncertainty was determined using ProNP3 (PROLAB) software.

## Results and discussion

In this study 163 pesticides (6 acaricides, 62 fungicides, 18 herbicides and 77 insecticides) which may be found in herb samples were investigated using the MSPD and LSE procedures. Because these target analytes represent various substance groups (Table 2) with different physico-chemical properties, development of a simple and reliable multiresidue analytical method to determine pesticide residues in a complex herb matrix was a considerable challenge.

**Table 2:** MSPD validation parameters for 163 active substances of four different herbs (sorted by substance group)

No.	Substance group	Active substance	Pesticide type	Detector	*R* ^2^	LOD (mg/kg)	LOQ (mg/kg)	Linden (*Tilia*)		Lungwort (*Pulmonaria* L.)		Melissa (*Melissa* L.)		Peppermint (*Mentha piperita* L.)	
								Rec. (*n* = 3) (%)	RSD (%)	Rec. (*n* = 3) (%)	RSD (%)	Rec. (*n* = 3) (%)	RSD (%)	Rec. (*n* = 3) (%)	RSD (%)
1.	Acylalanine	Benalaxyl	F	NP	0.99986	0.010	0.020	105	8	104	8	102	2	102	4
2.	Alkanamide	Napropamide	H	NP	0.99983	0.010	0.020	95	7	99	9	105	14	100	8
3.	Amine	Diphenylamine	F	NP	0.99998	0.010	0.020	86	5	105	3	83	3	100	4
4.	Anilinopyrimidine (3)	Cyprodinil	F	NP	0.99600	0.005	0.007	105	7	103	6	106	5	107	5
5.		Mepanipyrim	F	NP	0.99994	0.010	0.020	97	6	98	10	101	2	103	3
6.		Pyrimethanil	F	NP	0.99980	0.005	0.007	84	10	96	12	99	8	97	10
7.	Aryloxyphenoxypropionate	Fluazifop-p-butyl	H	NP	0.99738	0.010	0.020	88	13	98	5	92	9	87	6
8.	Benzamide	Propyzamide	H	EC/NP	0.99998	0.010	0.020	85	5	100	6	111	8	106	7
9.	Benzilate	Bromopropylate	A	EC	0.99988	0.005	0.010	91	8	115	4	107	2	105	6
10.	Benzonitrile	Dichlobenil	H	EC/NP	0.99994	0.005	0.010	72	9	105	2	90	11	103	4
11.	Bridged diphenyl	Tetradifon	A	EC	0.99686	0.007	0.010	74	2	95	8	103	6	93	8
12.	Carbamate (7)	Carbaryl	I	NP	0.99545	0.020	0.030	70	9	103	16	96	1	91	11
13.		Carbofuran	I, A	NP	0.99876	0.010	0.020	77	6	95	10	86	3	85	9
14.		Chlorpropham	H	NP	0.99997	0.008	0.010	103	7	108	3	104	7	101	6
15.		Iprovalicarb	F	NP	0.99998	0.010	0.020	82	5	104	5	106	8	100	3
16.		Pirimicarb	I	NP	1.00000	0.005	0.008	90	6	94	9	98	10	91	7
17.		Propham	H	NP	0.99999	0.008	0.010	78	8	105	12	109	6	103	10
18.		Propoxur	I, A	NP	0.99908	0.008	0.010	80	7	92	14	101	7	92	11
19.	Carboxamide (2)	Boscalid	F	EC/NP	0.99953	0.005	0.007	83	10	96	5	97	9	89	3
20.		Hexythiazox	A	EC/NP	0.99976	0.030	0.040	82	5	96	2	91	8	86	5
21.	Chlorinated aromatic hydrocarbon	HCB	F	EC	0.99998	0.005	0.007	101	9	96	4	107	13	93	11
22.	Chlorinated hydrocarbon	Dieldrin	I	EC	0.99984	0.004	0.008	80	4	87	13	86	14	77	11
23.	Chloroacetamide (3)	Acetochlor	H	EC/NP	0.99996	0.010	0.015	108	9	103	5	102	7	106	6
24.		Metazachlor	H	EC/NP	0.99970	0.009	0.010	71	8	77	7	74	3	80	4
25.		Propachlor	H	EC/NP	0.99989	0.010	0.015	105	7	110	9	105	10	104	7
26.	Chloronitrile	Chlorothalonil	F	EC/NP	0.99653	0.008	0.010	89	9	85	11	95	15	100	5
27.	Chlorophenyl (5)	Dicloran	F	EC	0.99568	0.008	0.010	74	14	91	16	86	14	94	3
28.		Quintozene	F	EC	0.99942	0.004	0.006	81	4	109	15	72	11	99	2
29.		Tecnazene	F	EC	0.99743	0.005	0.009	98	2	115	2	84	4	104	5
30.		Tetrachlorvinphos	I, A	EC/NP	0.99969	0.008	0.010	92	16	92	3	91	9	96	4
31.		Tolclofos-methyl	F	EC/NP	0.99983	0.005	0.009	83	9	103	5	116	5	93	6
32.	Cyanoacetamide ozime	Cymoxanil	F	EC/NP	0.99866	0.040	0.050	98	5	108	7	96	6	102	7
33.	Cyclodiene	Aldrin	I	EC	0.99947	0.003	0.005	83	8	86	5	82	13	85	8
34.	Dicarboximide (2)	Iprodione	F	EC/NP	0.99967	0.008	0.010	108	8	101	9	115	15	99	6
35.		Procymidone	F	EC/NP	0.99939	0.006	0.010	104	4	113	3	108	5	106	4
36.	Dinitroaniline (2)	Pendimethalin	H	EC/NP	0.99999	0.008	0.010	99	9	103	6	79	8	102	4
37.		Trifluralin	H	EC/NP	0.99996	0.007	0.010	108	2	101	2	99	3	100	7
38.	Diphenyl ether	Nitrofen	H	EC	0.99913	0.005	0.007	109	3	99	1	89	12	95	3
39.	Hydroxyanilide	Fenhexamid	F	EC/NP	0.99742	0.009	0.010	96	1	108	1	106	8	89	5
40.	Imidazole (3)	Fenamidone	F	EC/NP	0.99999	0.010	0.020	74	7	98	1	93	7	98	4
41.		**Imazalil**	F	EC	0.99254	0.009	0.010	*53*	*3*	*55*	*12*	*66*	*4*	*67*	*8*
42.		Prochloraz	F	EC/NP	0.99706	0.008	0.010	84	2	79	10	73	8	72	9
43.	Isoxazolidinone	Clomazone	H	NP	1.00000	0.020	0.030	81	8	98	5	95	4	82	6
44.	Morpholine (2)	Dimethomorph	F	NP	0.99507	0.010	0.020	80	7	100	1	93	12	97	4
45.		Fenpropimorph	F	NP	0.99991	0.010	0.020	84	4	104	14	96	4	100	10
46.	Neonicotinoid	**Acetamiprid**	I	EC/NP	0.99562	0.009	0.010	*48*	*5*	*51*	*15*	*45*	*5*	*55*	*10*
47.	Organochlorine (15)	Alpha-HCH	I	EC	0.99461	0.004	0.005	96	4	93	3	92	6	87	10
48.		Beta-HCH	I	EC	0.99963	0.007	0.009	94	9	93	2	97	6	88	9
49.		Dicofol	A	EC	0.99937	0.005	0.008	107	7	105	15	114	4	118	1
50.		Endrin	I	EC	0.99934	0.004	0.005	82	1	89	3	87	2	85	8
51.		Gamma-HCH (lindane)	I, A	EC	0.99898	0.005	0.007	102	10	103	11	101	3	109	8
52.		Endosulfan-alpha	I, A	EC	0.99956	0.005	0.008	84	14	99	8	95	5	92	12
53.		Endosulfan-beta	I, A	EC	0.99794	0.005	0.008	82	6	99	3	93	9	83	5
54.		Endosulfan-sulfate	I, A	EC	0.99779	0.005	0.008	88	6	94	14	96	2	91	14
55.		Heptachlor	I	EC	0.99998	0.004	0.005	92	5	97	4	99	4	96	3
56.		Heptachlor-epoxide	I	EC	0.99997	0.004	0.005	89	2	101	7	107	2	91	16
57.		Methoxychlor (DMDT)	I	EC	0.99967	0.006	0.010	116	2	114	9	113	3	117	8
58.		op’ -DDT	I	EC	0.99989	0.004	0.005	76	2	77	5	70	9	72	3
59.		pp’ -DDD	I	EC	0.99995	0.004	0.005	86	2	94	2	95	2	90	7
60.		pp’ -DDE	I	EC	0.99890	0.003	0.004	91	14	95	8	91	9	96	6
61.		pp’ -DDT	I	EC	0.99920	0.006	0.007	71	9	75	12	80	6	73	7
62.	Organophosphate (31)	Azinphos-ethyl	I, A	EC/NP	0.99990	0.008	0.010	94	1	112	5	92	7	101	4
63.		Azinphos-methyl	I, A	EC/NP	0.99771	0.008	0.010	81	7	91	6	89	10	96	7
64.		Chlorfenvinphos	I, A	EC/NP	0.99989	0.007	0.010	85	8	109	13	94	7	99	11
65.		Chlorpyrifos	I	EC/NP	0.99964	0.005	0.007	97	8	100	10	101	8	98	6
66.		Chlorpyrifos-methyl	I, A	EC/NP	0.99999	0.005	0.007	105	7	107	8	103	5	100	5
67.		Coumaphos	I	EC/NP	0.99997	0.008	0.010	77	10	102	6	105	6	99	5
68.		Diazinon	I, A	EC/NP	0.99998	0.005	0.008	105	8	108	1	115	10	100	3
69.		**Dimethoate**	I, A	EC/NP	0.99999	0.008	0.010	*62*	*8*	*66*	*2*	*69*	*4*	*68*	*3*
70.		Ethion	I, A	EC/NP	0.99999	0.009	0.010	76	8	78	15	86	12	88	5
71.		Ethoprophos	I	EC/NP	0.99994	0.007	0.010	72	10	114	6	113	13	111	8
72.		Fenitrothion	I	EC/NP	0.99985	0.005	0.009	89	7	98	5	106	6	99	7
73.		**Fenthion**	I	NP	0.99997	0.009	0.010	*58*	*10*	*63*	*3*	*54*	*1*	*66*	*7*
74.		Heptenophos	I	NP	0.99998	0.008	0.010	78	4	87	15	96	7	83	11
75.		Isofenphos	I	EC/NP	1.00000	0.006	0.010	74	13	73	10	89	6	86	6
76.		Isofenphos-methyl	I	EC/NP	0.99992	0.006	0.010	106	9	108	2	109	16	100	3
77.		Malaoxon	I, A	NP	1.00000	0.005	0.009	92	15	103	1	108	7	98	2
78.		Malathion	I, A	EC/NP	0.99992	0.008	0.010	81	12	103	5	95	7	81	7
79.		Mecarbam	I, A	EC/NP	0.99995	0.008	0.010	88	5	85	8	97	11	87	9
80.		Methidathion	I, A	EC/NP	0.99994	0.007	0.010	81	2	102	9	98	16	92	8
81.		Paraoxon-ethyl	I	EC/NP	0.99997	0.005	0.010	74	8	74	6	90	2	84	5
82.		Paraoxon-methyl	I	EC/NP	0.99945	0.005	0.010	100	4	103	5	98	7	93	4
83.		Parathion	I, A	EC/NP	0.99932	0.008	0.010	96	6	101	10	109	10	91	11
84.		Parathion-methyl	I	EC/NP	0.99967	0.008	0.010	105	3	105	12	117	9	102	10
85.		**Phorate**	I, A	EC/NP	0.99995	0.009	0.010	*55*	*4*	*65*	*13*	*49*	*8*	*61*	*11*
86.		Phosalone	I, A	EC/NP	0.99981	0.007	0.010	94	7	103	5	99	13	97	10
87.		Phosmet	I, A	EC/NP	0.99987	0.009	0.010	96	3	104	8	102	7	100	6
88.		Pirimiphos-ethyl	I, A	NP	0.99999	0.005	0.008	74	4	70	1	82	3	76	2
89.		Pirimiphos-methyl	I, A	NP	0.99996	0.005	0.008	95	7	104	2	111	1	103	3
90.		Profenofos	I, A	EC/NP	0.99995	0.008	0.010	73	3	72	6	88	5	75	8
91.		Quinalphos	I, A	EC/NP	0.99608	0.005	0.006	75	1	107	3	113	16	107	5
92.		Triazophos	I, A	NP	0.99999	0.007	0.010	112	7	106	4	116	10	101	3
93.	Organothiophosphate (5)	Bromophos-ethyl	I	EC/NP	0.99956	0.005	0.007	86	5	104	8	100	14	84	9
94.		Bromophos-methyl	I	EC/NP	0.99996	0.005	0.007	93	5	104	5	97	7	94	3
95.		Fenchlorphos	I	EC/NP	0.99987	0.009	0.010	100	10	104	6	94	4	99	5
96.		**Formothion**	I, A	EC/NP	0.99984	0.008	0.010	*68*	*15*	*58*	*8*	*65*	*10*	*59*	*9*
97.		Methacrifos	I, A	EC/NP	0.99979	0.009	0.010	82	7	94	10	95	9	84	12
98.	Oxadiazine	Indoxacarb	I	EC/NP	0.99965	0.009	0.010	93	8	101	12	91	7	91	11
99.	Oxazole	Vinclozolin	F	EC/NP	0.99971	0.009	0.010	92	8	104	13	94	4	102	9
100.	Phenylamide (2)	Metalaxyl	F	NP	0.99986	0.008	0.010	89	9	95	5	103	5	97	4
101.		Oxadixyl	F	EC/NP	0.99995	0.020	0.030	70	10	75	6	77	3	71	5
102.	Phenylpyrazole	Fipronil	I	EC/NP	0.99988	0.004	0.005	84	4	83	9	92	5	83	7
103.	Phenylpyrrole	Fludioxonil	F	NP	0.99908	0.008	0.010	100	5	107	5	87	4	88	6
104.	Phosphorothiolate	Pyrazophos	F	EC/NP	1.00000	0.009	0.010	73	7	76	14	76	16	79	11
105.	Phthalimide (2)	**Captan**	F	EC	0.99917	0.008	0.010	*59*	*13*	*68*	*16*	*50*	*12*	*67*	*6*
106.		**Folpet**	F	EC	0.99889	0.008	0.010	*69*	*15*	*64*	*12*	*56*	*10*	*59*	*10*
107.	Pyrazole	Tebufenpyrad	A	NP	0.99998	0.009	0.010	103	11	106	9	105	8	102	6
108.	Pyrethroid (13)	Acrinathrin	I, A	EC	0.99976	0.010	0.020	106	5	96	6	108	2	98	9
109.		Alpha-cypermethrin	I	EC/NP	0.99892	0.006	0.010	93	13	99	6	100	10	95	7
110.		Beta-cyfluthrin	I	EC/NP	0.99962	0.009	0.010	86	2	101	8	99	9	88	9
111.		Bifenthrin	I, A	EC	0.99980	0.009	0.010	94	8	105	3	96	6	94	9
112.		Cyfluthrin	I	EC/NP	0.99920	0.009	0.010	74	6	94	6	96	13	93	5
113.		Cypermethrin	I	EC	0.99993	0.009	0.010	73	16	85	12	81	5	86	11
114.		Deltamethrin	I	EC/NP	0.99993	0.009	0.010	87	9	93	5	94	8	89	8
115.		Esfenvalerate	I	EC/NP	0.99044	0.008	0.010	76	5	104	3	94	11	84	4
116.		Fenpropathrin	I, A	EC/NP	0.99997	0.007	0.010	80	10	94	5	88	7	79	8
117.		Fenvalerate	I, A	EC/NP	0.99965	0.008	0.010	74	3	97	14	83	7	87	12
118.		Lambda-cyhalothrin	I	EC/NP	0.99933	0.008	0.010	77	5	94	16	74	5	92	15
119.		Permethrin	I	EC	0.99983	0.009	0.010	75	10	97	10	101	2	90	11
120.		Zeta-cypermethrin	I	EC	0.99977	0.008	0.010	97	5	98	6	102	8	96	7
121.	Pyridazinone	Pyridaben	I, A	EC/NP	0.99992	0.010	0.020	77	2	80	9	100	4	83	8
122.	Pyrimidine	Fenarimol	F	EC/NP	0.99995	0.009	0.010	76	4	85	7	88	9	81	8
123.	Pyrimidinol	Bupirimate	F	EC/NP	0.99974	0.008	0.010	88	8	97	8	118	8	97	7
124.	Quinoline	Quinoxyfen	F	EC/NP	0.99996	0.009	0.010	103	1	99	2	107	3	100	3
125.	Strobilurin (5)	Azoxystrobin	F	EC/NP	0.99960	0.009	0.010	91	6	105	5	107	7	97	4
126.		Dimoxystrobin	F	EC/NP	0.99999	0.008	0.010	78	5	92	6	92	11	96	5
127.		Kresoxim-methyl	F	EC/NP	0.99994	0.007	0.010	99	2	108	4	103	5	97	8
128.		Pyraclostrobin	F	EC/NP	1.00000	0.010	0.020	84	2	82	10	108	16	81	11
129.		Trifloxystrobin	F	EC/NP	0.99993	0.005	0.008	78	2	83	6	104	6	85	8
130.	Strobilurin type-methoxyacrylate	Picoxystrobin	F	EC/NP	0.99998	0.008	0.010	77	11	79	9	96	3	77	9
131.	Sulphamide (2)	Dichlofluanid	F	EC/NP	0.99407	0.006	0.010	77	5	98	6	74	6	89	8
132.		Tolylfluanid	F	EC/NP	0.99987	0.008	0.010	76	2	89	1	74	2	99	2
133.	Triazine (3)	Atrazine	H	NP	1.00000	0.007	0.010	107	7	100	5	96	1	103	6
134.		Prometrine	H	NP	0.99998	0.009	0.010	72	3	104	6	93	2	84	9
135.		Simazine	H	NP	0.99999	0.006	0.010	77	8	101	5	113	1	91	7
136.	Triazinone	Metribuzin	H	EC/NP	0.99979	0.008	0.010	76	8	96	2	81	2	94	3
137.	Triazole (21)	Azaconazole	F	EC/NP	0.99995	0.009	0.010	73	8	78	2	81	5	82	4
138.		Biterthanol	F	NP	0.99959	0.009	0.010	103	3	104	5	75	5	77	5
139.		Cyproconazole	F	EC/NP	0.99999	0.009	0.010	97	8	103	6	101	3	90	8
140.		Difenoconazole	F	EC/NP	0.99996	0.008	0.010	75	7	72	3	77	8	72	4
141.		Diniconazole	F	EC/NP	0.99972	0.007	0.010	86	1	87	6	110	11	87	5
142.		Epoxiconazole	F	EC/NP	0.99999	0.009	0.010	96	10	90	5	111	2	95	6
143.		Fenbuconazole	F	NP	0.99976	0.009	0.010	90	5	91	7	98	4	95	5
144.		Fluquinconazole	F	EC/NP	0.99975	0.009	0.010	74	8	75	6	106	5	87	7
145.		Flusilazole	F	NP	0.99985	0.007	0.010	78	1	107	3	115	9	102	6
146.		Flutriafol	F	EC/NP	0.99994	0.009	0.010	71	3	88	4	86	10	76	5
147.		Hexaconazole	F	EC/NP	0.99971	0.006	0.010	100	9	104	5	92	8	101	3
148.		Imibenconazole	F	EC/NP	0.99990	0.008	0.010	87	10	86	6	98	3	84	9
149.		Metconazole	F	NP	0.99993	0.008	0.010	95	7	105	8	93	5	90	9
150.		Myclobutanyl	F	EC/NP	0.99992	0.009	0.010	101	9	106	10	109	4	103	11
151.		Paclobutrazol	F	EC/NP	0.99995	0.015	0.020	94	4	110	5	114	8	101	6
152.		Penconazole	F	EC/NP	0.99946	0.009	0.010	95	6	109	6	104	8	91	7
153.		Propiconazole	F	EC/NP	0.99977	0.008	0.010	92	8	99	4	105	5	94	8
154.		Tebuconazole	F	NP	0.99990	0.007	0.010	100	9	103	2	110	7	109	3
155.		Tetraconazole	F	EC/NP	0.99987	0.008	0.010	101	4	102	6	106	8	100	7
156.		Triadimefon	F	EC/NP	0.99998	0.008	0.010	88	6	90	4	92	5	95	5
157.		Triadimenol	F	EC/NP	1.00000	0.009	0.010	106	11	106	6	105	11	101	7
158.	Triazole (isomer mix)	Bromuconazole	F	EC/NP	0.99996	0.008	0.010	97	13	104	2	93	8	91	3
159.	Unclassified (4)	Fenazaquin	A, I	NP	0.99990	0.010	0.015	102	5	100	2	102	3	104	3
160.		Buprofezin	I,A	EC/NP	0.99995	0.008	0.009	81	2	85	5	88	8	85	6
161.		DEET	I	NP	0.99999	0.015	0.020	106	4	106	6	100	9	106	7
162.		Pyriproxyfen	I	NP	0.99995	0.020	0.025	73	11	78	5	71	10	78	6
163.	Uracyl	Lenacil	H	NP	0.99998	0.010	0.020	74	5	75	4	76	4	75	5

Pesticides in *bold* have recovery below 70 % (values in *italics*)

*A* acaricide, *F* fungicide, *H* herbicide, *I* insecticide, *EC* electron capture, *NP* nitrogen phosphorus, *R* ^2^ correlation coefficient, *RSD* relative standard deviation, *Rec*. mean recovery

### Optimization of extraction techniques

The studies were carried out by varying different parameters: sorbent, sample to sorbent mass ratio, extracting solvent, extraction time and clean-up sorbent. Conditions for the best extraction efficiency were used for the rest of the study.

Preliminary studies were performed to evaluate the efficiency of MSPD. Various sorbents such as Florisil and silica gel, activated and deactivated, were tested. The use of deactivated sorbents gave recoveries below 40 %. The optimum extraction conditions were obtained with activated Florisil (activation temperature 600 °C). The determination of the plant matrix to sorbent (Florisil) mass ratio was the second step of the optimization procedure. The following mass ratios were examined: 1:1, 1:2, 1:4; a herb to sorbent mass ratio of 1:2 was found to be the most satisfactory. The selection of a dispersing solvent and its volume was the third step of the MSPD optimization procedure. Acetone, diethyl ether, hexane, methanol and its mixtures in different ratios were tested in this step. The most appropriate extraction solvent was acetone/methanol (9:1, v/v). The experiment revealed that 25 ml of the mixture was sufficient for effective elution of pesticide residues. Although 10 ml of methanol produced similar yields, it did not evaporate as quickly as the solvent mixture mentioned above.

**Fig. 2:**
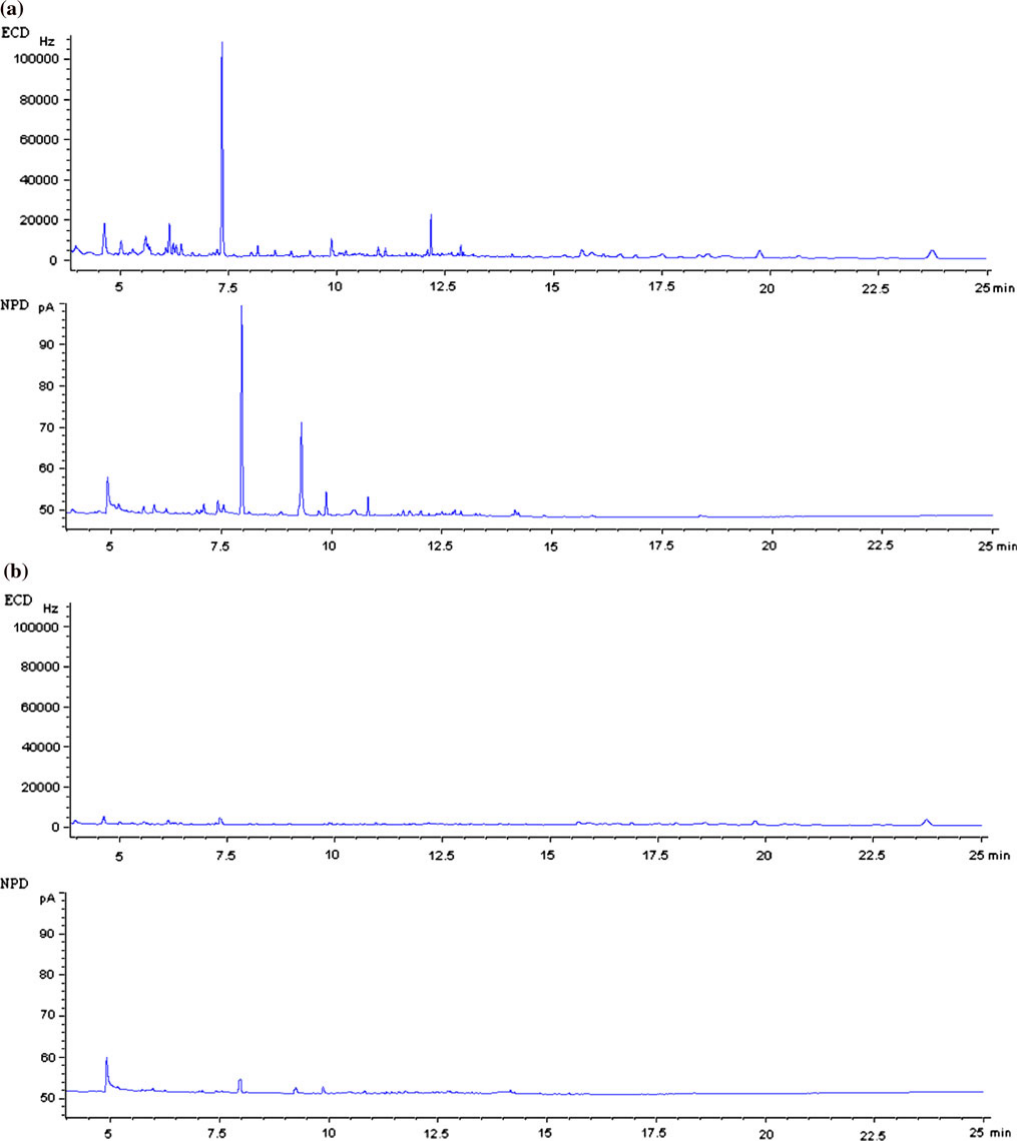
Chromatogram of blank linden sample obtained from MSPD extract: **a** without C_18_; **b** with C_18_

Our experiments showed that the final MSPD extract contained a large amount of matrix co-extracts (Fig. 2a). These can impact the analyte identification by GC-NP/EC. Interfering peaks with retention times close to those of the target residue are the main factors which reduce the achievement of low detection limits. To protect the GC system as much as possible, we focused on reducing the level of the co-extracted matrix. Several cleaning sorbents such as PSA, GCB and C_18_ were tested. The addition of 1 g of octadecyl C_18_ as a clean-up sorbent at the bottom of the chromatography column was necessary to minimize interference and produced the best recoveries. The optimum extraction conditions with high recovery were conducted with a 2 g herb sample and 4 g of Florisil as a sorbent, along with a simultaneous stage of clean-up with C_18_. A chromatogram of a blank linden sample where octadecyl sorbent was used for the preparation of a MSPD extract is shown in Fig. 2b. The MSPD method proposed for the analysis of pesticides in herbs provided clean blank extracts and therefore no additional clean-up step was necessary.

In preliminary tests with LSE, the solvent and extraction time were tested. Acetone, acetonitrile, hexane, diethyl ether and their mixtures were tested. During experiments we found that a decrease of solvent polarity (acetonitrile → hexane) led to reduced solubility of polar co-extracts in the hexane extract. Unfortunately, poor recoveries were obtained for polar pesticides. Finally, most of the pesticides were recovered from a 2 g sample shaken with 70 ml of hexane/diethyl ether/acetone (1:2:2, v/v/v) (50 ml and an additional 20 ml portion). An increase in the extraction mixture volume up to 100 ml resulted in no significant improvement in analyte recoveries. Additionally, pesticide recoveries increased when the extraction time was extended to 1 h; however, further increase in the extraction time to 2 h provided slightly lower values. Therefore, 1 h was selected as the extraction time for this procedure.

**Fig. 3:**
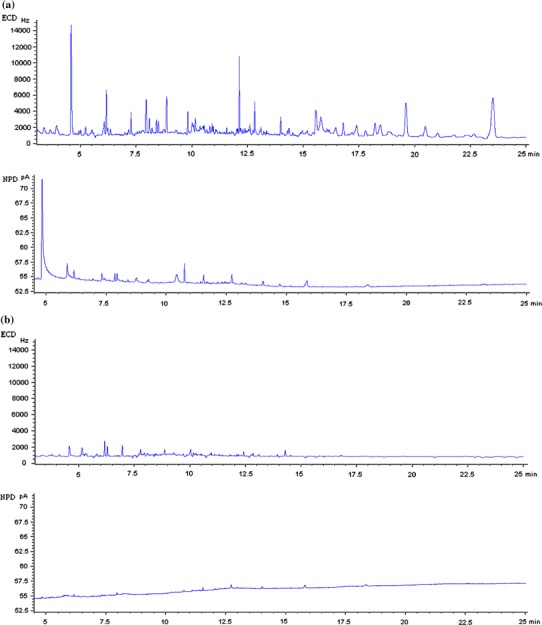
Chromatogram of blank linden sample extract obtained from liquid–solid extraction (LSE): **a** before clean-up; **b** after clean-up

Due to the presence of interfering peaks from the matrix, further clean-up stages were necessary (Fig. 3a) to reduce the amounts of matrix co-extracts. Purification of the extract was carried out using a chromatography column packed with sodium sulfate (1 g), 5 % neutral aluminum oxide (2 g) and 4 % Florisil (2 g). Analyte recoveries were calculated against extraction volume at different hexane/dichloromethane ratios: 9:1, 8:2 and 7:3. The best results were achieved using 30 ml of a mixture of hexane/dichloromethane (7:3, v/v) (Fig. 3b).

In summarizing the above optimization steps for both procedures, we observed that the MSPD extraction offers important savings in time (extraction up to 15 min), requires less volume and toxic solvent for efficient isolation of analyzed compounds and was faster and simpler to perform when compared with LSE.

### Comparison of extraction techniques

Considering the amounts of co-isolated matrix compounds, but also the recovery of the target analytes as an important performance characteristic of the analytical method, MSPD extraction was investigated for the extraction of multiple pesticide residues (163) from herb samples at spiking levels ranging from 0.05 to 0.5 mg/kg in a subsequent experiment.

**Fig. 4:**
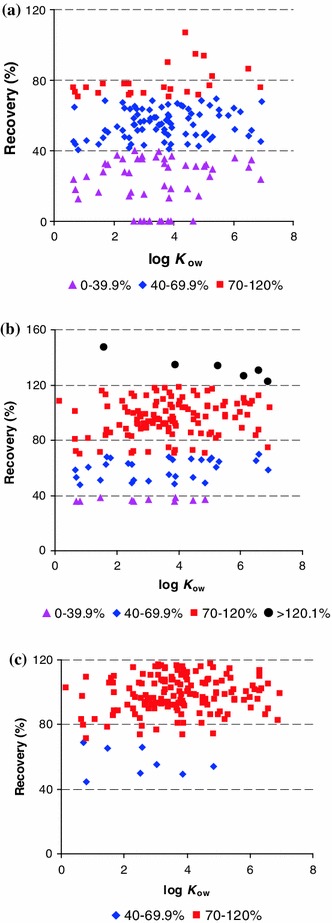
Recoveries (%) of pesticides tested vs. their log*K*_ow_. **a** LSE hexane extract **b** LSE hexane/diethyl ether/acetone extract **c** MSPD

The pesticides studied covered a wide range of polarities, from the polar propoxur (log*K*_ow_ = 0.14) to non-polar lambda-cyhalothrin (log*K*_ow_ = 6.9). Data in Fig. 4 for both methods were obtained from linden samples spiked at levels ranging from 0.05 to 0.5 mg/kg. The values of octanol–water partition coefficients were found in databases [35, 36]. Comparing the results obtained in Fig. 4, it can be observed that MSPD successfully recovered 155 pesticides, with recoveries >70 %, whereas LSE was effective with only 24 pesticides (hexane extract) and 118 pesticides (hexane/diethyl ether/acetone extract). As shown by Fig. 4a, pesticides with log*K*_ow_ <4 were poorly extracted through the use of LSE. Lower recoveries of these pesticides may be explained by the use of a non-polar solvent (hexane) required for the elimination of most of the matrix co-extracts. On the other hand, liphophilic pesticides (log*K*_ow_ >4) had acceptable recoveries (>40 %) but they represent only 33 % of all compounds analyzed. Better recoveries were obtained using more polar mixtures of solvents: hexane/diethyl ether/acetone (1:2:2, v/v/v) Fig. 4b. However, the results was not satisfactory enough because recoveries in the range 70–120 % comprised 72 % pesticides of all tested, and many active substances resulted in recoveries <70 and >120 %.

Using the MSPD method represents the best choice for most pesticides (Fig. 4c): satisfactory recoveries (70–120 %) for most pesticides were obtained with this method. Several exceptions (acetamiprid, captan, dimethoate, fenthion, folpet, formothion, imazalil and phorate) (<70 %) were observed. Finally, the MSPD extraction technique provided better results in terms of recovery of target analytes and the amount of isolated matrix co-extracts.

### Matrix effect

The response of the detectors to certain pesticides may be affected by the presence of co-extractives from the sample. These matrix effects may be observed as an increase or decrease in response compared with those produced by solvent solutions of the analyte. The effect of the matrix can be variable and unpredictable in the occurrence of measurable effects. The matrix effect on the detector (EC and NP) response for the pesticides and matrices studied was evaluated in the present work. To determine if there was a different response between matrix-matched standards and standards in solvent, matrix-matched standards were used.

### Validation for the analysis of pesticides

The MSPD optimization procedure was investigated to determine conditions which would be general for various herbs. The procedure involving MSPD extraction was validated with six different herb samples fortified at three spiking levels: the first ranging between 0.005 and 0.05 mg/kg, the second at 0.05–0.5 mg/kg and the third at 0.25–2.5 mg/kg (HP-5 column).

The GC-NP/EC analytical conditions in this study allow for the analysis of all target compounds in a single chromatographic run of 25.431 min. All pesticides were satisfactorily separated with high sensitivity and selectivity.

The applicability of the MSPD for different kinds of herbs was examined in the present experiment. The validation parameters of linden, lungwort, melissa and peppermint are given in Table 2.

Response linearity of the method was found in the concentration range studied, with correlation coefficients between 0.99254 and 1.00000. Calibration curves were obtained from matrix matching calibration solutions. The precision of the method was evaluated and expressed as RSD (%) at three concentration levels. Table 2 shows the results with RSD values (RSDs ≤16 %). Accuracy was also evaluated at three concentration levels. As seen in Table 2, the mean recovery values were in the range of 70–119 % for most pesticides. There were several exceptions: acetamiprid, captan, dimethoate, fenthion, folpet, formothion, imazalil and phorate, where recoveries were below 70 %. Most results for MSPD were within the acceptable range (70–120 %) and indicate that this method was both accurate and precise. LODs and LOQs of all tested pesticide residues extracted using the MSPD technique and analyzed through GC-EC/NP were determined in order to evaluate the efficiency and availability of the method. The LODs and LOQs ranged from 0.003 to 0.03 mg/kg and 0.005 to 0.04 mg/kg, respectively.

**Fig. 5:**
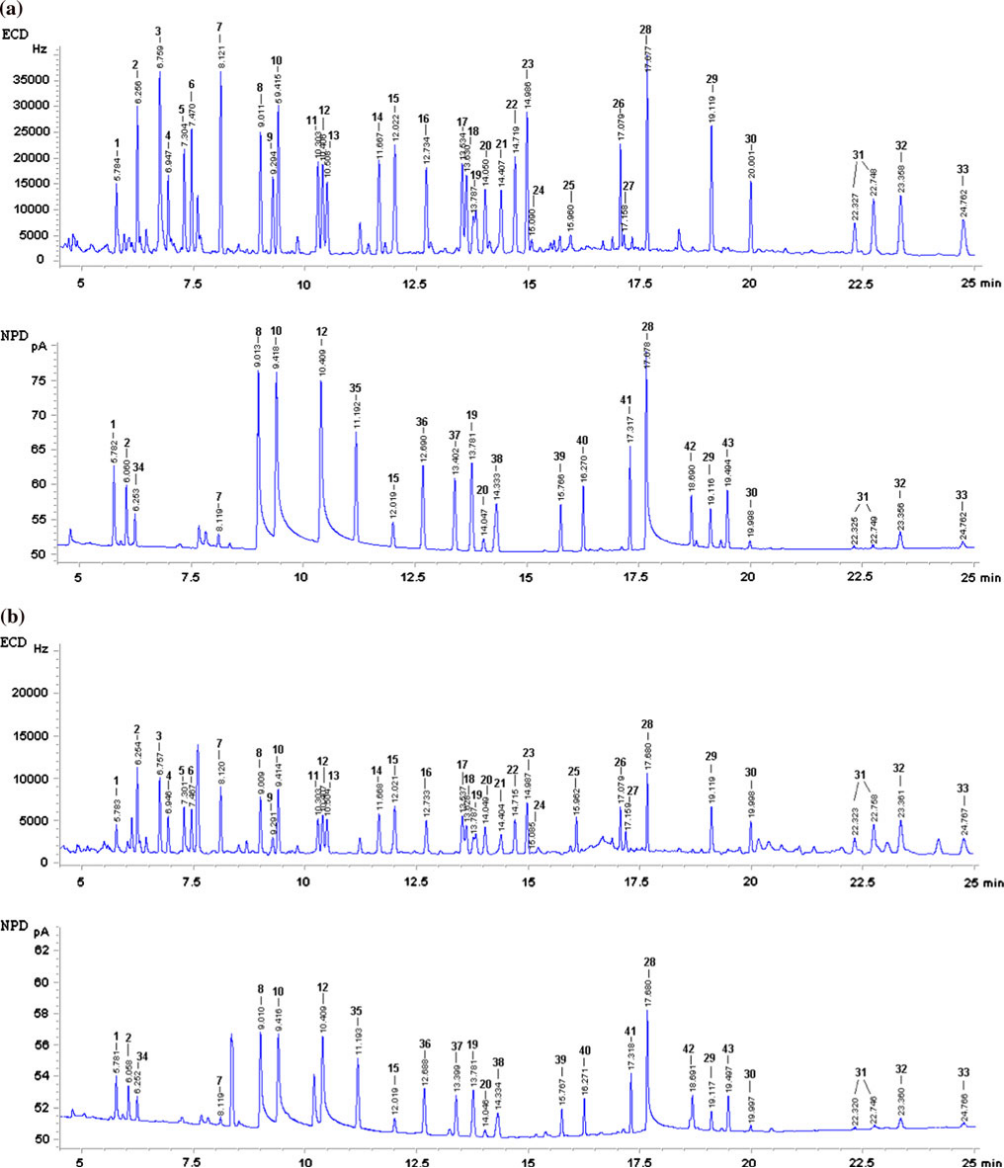
Chromatogram of: **a** selected multicompound standard mixture in matrix; **b** linden sample fortified with selected multicompound standard mixture: *1* propachlor, *2* trifluralin, *3* alpha-HCH, *4* HCB, *5* beta-HCH, *6* gamma-HCH, *7* chlorothalonil, *8* chlorpyrifos methyl, *9* heptachlor, *10* fenchlorphos, *11* aldrine, *12* chlorpyrifos, *13* dicofol, *14* heptachlor epoxide, *15* procymidone, *16* alpha-endosulfan, *17* pp′-DDE, *18* dieldrin, *19* myclobutanyl, *20* krezoxim-methyl, *21* endrin, *22* beta-endosulfan, *23* pp′-DDD, *24* op′-DDT, *25* pp′-DDT, *26* bifenthrin, *27* DMDT, *28* phosalone, *29* prochloraz, *30* boscalid, *31* deltamethrin (isomers), *32* azoxystrobin, *33* imibenconazole, *34* chlorpropham, *35* cyprodinil, *36* mepanipyrim, *37* fludioxonil, *38* cyproconazole, *39* benalaxyl, *40* tebuconazole, *41* fenazaquin, *42* bitertanol, *43* fenbuconazole

The above results prove that MSPD fulfilled the requirements in all herbs tested. MSPD was found to be adequate for the analysis of herbs with differing amounts of essential oil components. Chromatograms of a selected multicompound standard mixture (containing 43 active substances) in the matrix and a linden sample spiked with this mixture (extracted using MSPD) are presented in Fig. 5a, b, respectively.

The different aspects explained above for estimating the standard uncertainties were applied to the multiresidue analytical method. A methodology for calculating the uncertainty of results on the basis of in-house validation data was applied to the pesticide multiresidue method. Uncertainty sources were identified and standard uncertainty was established. An increase in the uncertainty when reducing the level of concentration of the active substance in the sample was observed. However, depending on the concentration and the physico-chemical parameters of the active substance determined, the combined standard uncertainty of the MSDP method for all compounds ranged from 15 to 30 %.

### Quality control

The laboratory was accredited in accordance with the PN-EN ISO/IEC 17025:2005 standards [37] in 2007 and participates in proficiency testing schemes organized and run by the Food Analysis Performance Assessment Scheme (FAPAS; Central Science Laboratory in York) and by the European Commission (initially by the University of Uppsala and then by the University of Almeria) each year. Additionally, the MSPD method developed was accredited and the procedure for pesticide residue determination in herbs was incorporated into the scope of the laboratory accreditation in 2010.

### Real samples

In the final phase of this work, the validated MSPD method was used for routine pesticide analysis of 15 herb samples to evaluate its performance and applicability. The samples analyzed included chamomile, linden, lungwort, melissa, peppermint and thyme. No pesticide residues were found in 87 % of the samples. The positive results were confirmed using columns of different polarity. Chlorpyrifos was found in the melissa sample with a concentration of 0.21 mg/kg above the maximum residue limit (MRL = 0.5 mg/kg according to Regulation (EC) 396/2005 [10]) and pp′-DDD was found in the linden sample 0.02 mg/kg below MRL (MRL = 0.5 mg/kg). It is necessary to point out that pp′-DDD was found in one sample, and that this pesticide belongs to the chlorinated pesticide group and is a product of the breakdown of DDT, a pesticide banned for agricultural use worldwide under the Stockholm Convention [38]. Our results showed that regular monitoring of herb samples for pesticide residues is necessary to protect human health.

## Conclusions

In this study we tested two preparation techniques and presented a novel solution for the rapid analysis of multiple pesticide residues in herbs. A fast and simple MSPD method was developed to detect the residues of 163 pesticides in herbs using gas chromatography. This method showed a high sensitivity and the confirmatory power necessary for the determination of pesticide residues at the levels required by the European MRL for herbs. The proposed method not only allowed the simultaneous determination and confirmation of a very large number of pesticides acceptable in terms of recovery and detection limits, but was also shown to be useful in routine analysis since it is fast and easy to carry out. The extraction procedures evaluated allowed for determination of pesticides from different classes: carbamate (7), organochlorine (15), organophosphate (31), organothiophosphate (5), pyrethroid (13), strobilurin (5) and triazole (21), as well as those belonging to other substance groups often used in plant protection products.

There is undoubtedly a tendency to replace some MSPD extraction methods for pesticide analysis in food matrices with QuEChERS, but this choice seems to be dictated more by prejudice than by evidence, so studies comparing the two techniques and a more accurate choice of material for MSPD would be useful [39].

In our study, MSPD, in comparison with LSE, is an inexpensive and simple sample preparation procedure allowing the reduction of organic solvent consumption, significant savings in time, exclusion of sample component degradation, improvement of extraction efficiency and selectivity, and the elimination of the additional sample clean-up and pre-concentration steps before chromatographic analysis. MSPD has been demonstrated to be a suitable preparation technique for the isolation of pesticides from herbs when compared with classic multiresidue methods. For these reasons, the MSPD extraction technique fulfilled requirements of being a multiresidue method and enabled the isolation of all target pesticides with good validation parameters. Good quality control and determination of the presence of toxic pesticides in herbs is essential to avoid their overconsumption and cumulative toxicities in long-term use.
